# The role of calpains in ventilator-induced diaphragm atrophy

**DOI:** 10.1186/s40635-017-0127-4

**Published:** 2017-03-14

**Authors:** Xiaoping Zhu, Hieronymus W. H. van Hees, Leo Heunks, Feifei Wang, Lei Shao, Jiaru Huang, Lei Shi, Shaolin Ma

**Affiliations:** 1 Department of Pulmonary Diseases, Shanghai East Hospital, Tongji University, Shanghai, 200120 China; 2 Department of Pulmonary Diseases, Radboud University Medical Centre, Postbox 9101, Nijmegen, 6500 HB the Netherlands; 3 Intensive Care Medicine, Radboud University Medical Centre, Postbox 9101, Nijmegen, 6500 HB the Netherlands; 4 NingXia Medical University, Yinchuan, 750004 China; 5 Department of Intensive Care Unit, Shanghai East Hospital, Tongji University, Shanghai, 200120 China

**Keywords:** Calpain, Calpeptin, Mechanical ventilation, Diaphragm atrophy

## Abstract

**Background:**

Controlled mechanical ventilation (CMV) is associated with diaphragm dysfunction. Dysfunction results from muscle atrophy and injury of diaphragm muscle fibers. Enhanced proteolysis and reduced protein synthesis play an important role in the development of atrophy. The current study is to evaluate the effects of the calpains inhibitor calpeptin on the development of diaphragm atrophy and activation of key enzymes of the ubiquitin-proteasome pathway in rats under CMV.

**Methods:**

Three groups of rats were studied: control animals (CON, *n* = 8), rats subjected to 24 h of MV (CMV, *n* = 8), and rats subjected to 24 h of MV after administration of the calpain inhibitor calpeptin (CMVC, *n* = 8). The diaphragm was analyzed for calpain activity, myosin heavy chain (MHC) content, and cross-sectional area (CSA) of diaphragmatic muscle fibers as a marker for muscle atrophy. In addition, key enzymes of the ubiquitin-proteasome pathway (MAFbx and MuRF1) were also studied.

**Results:**

CMV resulted in loss of both MHC_fast_ and MHC_slow_. Furthermore, the CSA of diaphragmatic muscle fibers was significantly decreased after 24 h of CMV. However, calpain inhibitor calpeptin prevented loss of MHC and CSA after CMV. In addition, calpeptin prevented the increase in protein expression of calpain1 and calpain2 and reduced calpain activity as indicated by reduced generation of the calpain cleavage product αII-spectrin in the diaphragm. CMV-induced upregulation of both MAFbx and MuRF1 protein levels was attenuated by treatment with calpeptin.

**Conclusions:**

The calpain inhibitor calpeptin prevents MV-induced muscle atrophy. In addition, calpeptin attenuated the expression of key proteolytic enzymes known to be involved in ventilator-induced diaphragm atrophy, including MAFbx and MuRF1.

## Background

Mechanical ventilation is a life-saving intervention for patients with acute respiratory failure. However, it is recognized that mechanical ventilation may be associated with side effects, in particular lung injury [1] and respiratory muscle dysfunction often described as ventilator-induced diaphragm dysfunction (VIDD) [2, 3]. Respiratory muscle weakness in ICU patients is associated with prolonged weaning from mechanical ventilation and mortality [4]. Today, no specific treatment for ICU acquired respiratory muscle weakness is available. Improved understanding of the pathophysiology of ICU acquired respiratory muscle weakness may help to develop specific treatment strategies.

Studies in animal models and critically ill patients have demonstrated that VIDD results from both the loss of muscle proteins and dysfunction of the remaining contractile proteins [3, 5]. Loss of muscle proteins may result from increased protein degradation or inhibition of protein synthesis. The proteolytic ubiquitin-proteasome pathway (UPP) is the main proteolytic pathway in skeletal muscles [6] and involves several key enzymes including MAFbx and MuRF1. Activation of different components of the UPP in the diaphragm has been demonstrated in animals subjected to MV [7, 8] but also in ICU patients [3, 9]. The UPP cannot degrade intact myofibrils because neither the large myofibril nor thick and thin filaments could enter the central catalytic chamber of the proteasome. Activation of other enzymes, such as calpains cleaves major cytoskeletal proteins such as titin and nebulin, which leads to the release of myofilaments suitable for degradation by the UPP [10–12]. Today, only one study assessed the role of calpains in VIDD [12]. In that study, inhibition of calpain prevented MV-induced diaphragm atrophy.

The aim of the current study was to evaluate the effect of calpeptin, a specific calpain inhibitor, on ventilator-induced diaphragm atrophy and activation of proteolytic pathways.

## Methods

### Animals and experimental design

The study was approved by the University of Tongji Animal Care and Use Committee. Rats were offered by the Experimental Animal Center of Tongji Medical College. Twenty-four male Sprague-Dawley rats (body weight 320–350 g) were randomly assigned to one of three groups (*n* = 8/group): (1) acutely anesthetized control (CON), (2) 24 h of controlled mechanical ventilation (CMV), and (3) CMV rats treated with the calpain inhibitor calpeptin (CMVC). CMV rats received corresponding volumes of vehicle (0.1% DMSO in saline for calpeptin). Calpeptin (Sigma, MO, USA) was administered subcutaneously (4 mg/kg) 2 h before and 8, 15, and 23 h after initiation of mechanical ventilation. The timing and dosage of calpeptin administration 2 h before initiation of mechanical ventilation were in line with the study of Fareed MU [13]. Animals in the CON group were anesthetized with pentobarbital sodium (60 mg/kg body weight; intraperitoneal), and the diaphragm was removed immediately. Segments of the ventral costal region of the diaphragm were quickly removed, frozen in liquid nitrogen, and stored at −80 °C for subsequent biochemical analysis.

In ventilated rats, all surgical procedures were performed aseptic as previously described [14]. Animals selected for CMV were anesthetized with an intraperitoneal injection of pentobarbital sodium (60 mg/kg), tracheotomized and ventilated with a dedicated small-animal ventilator (TOPO; Kent Scientific, Connecticut, USA) for 24 h with the following settings: tidal volume 1 ml/100 g body weight, respiratory rate 80 breaths/min, and positive end-expiratory pressure 1 cm H_2_O. No visible diaphragm contractions were observed under mechanical ventilation. A time frame of 24 h was chosen based on previous data demonstrating significant diaphragm atrophy, proteolysis, and oxidative stress after this interval [15]. A catheter was inserted into the femoral vein for continuous infusion of isotonic saline (2 ml kg^−1^ h^−1^) and pentobarbital sodium (~10mg kg^−1^ h^−1^). Additionally, animals received enteral nutrition with a nutrient composition of 15% proteins, 35% lipids, 50% carbohydrates, and vitamins and minerals through an oral-gastric tube with total daily volume of 69 ml. Both heart rate and blood pressure were continuously monitored noninvasively with tail cuff. The care throughout the experimental period included lubricating the eyes, expressing the bladder, removing airway mucus and passively moving the limbs, and using glycopyrrolate (0.04 mg kg^−1^ 2h^−1^ intramuscular) to reduce bronchial secretions. Rectal temperature was monitored and maintained at 36–38 °C with a heating blanket. Arterial blood (100 μl per sample) from abdominal aorta was withdrawn at end of CMV and was analyzed for pH and the partial pressures of O_2_ and CO_2_ by an electronic blood-gas analyzer (radiometer, Copenhagen, Danmark). A research scientist provided round-the-clock coverage and animal care for the duration of the study. On completion of MV, segments of the ventral costal region were quickly removed and frozen in liquid nitrogen and stored at −80 °C for subsequent biochemical analysis, as described earlier [16].

### Biochemical measurements

Proteolytic activity of both calpain system and key enzymes of the UPP were analyzed by measuring protein levels of calpain1, calpain2, and the muscle-specific E3 ligases MAFbx and MuRF1 [7, 17]. In addition, calpain activities were evaluated by measuring the content of cleaved 145 kDa calpain fragment, which is specific to calpain1/2 cleavage products of αII-spectrin. Calpain activity was direct related to the expression level of 145-kDa cleavage product [18]. All of these proteins were measured by Western blotting.

### Western blotting

Section of the costal diaphragm was homogenized and assayed to quantitatively determine the levels of the proteins above. Tissue was homogenized 1:10 (5 mM Tris-HCl, pH = 7.5, 5 mM EDTA) and then the homogenate was centrifuged at 1500*g* for 10 min (4 °C). After determining total protein content, 50 μg of protein in samples were then individually separated by polyacrylamide gel electrophoresis. SDS-PAGE was performed on 6% (for MHC_slow_, MHC_2A_, and 145-kDa cleavage product) or 10% (for calpain1, calpain2, MuRF1, MAFbx, and glyceraldehyde-3-phosphate dehydrogenase (GADPH) polyacrylamide gels. After electrophoresis, the separated proteins were transferred electrophoretically using semi-dry transfer methodology to polyvinylidene fluoride membrane (Millipore). Membranes were stained with appropriate Marker (26616, Thermo Scientific) and visually inspected for equal protein loading and transfer. The membranes were then washed and blocked in 5% BSA for 1 h and subsequently incubated with primary antibodies directed against MHC_slow_, MHC_2A_, calpain1, calpain2, α-II spectrin 145-kDa cleavage product, MAFbx, MuRF1, and GAPDH. Primary antibodies were diluted 1:2000 (MHC_slow_, MHC_2A_, calpain1, calpain2) or 1:1,000 (145-kDa cleavage product, MAFbx, MuRF1) or 1:5000 (GAPDH) in blocking buffer and applied to the membranes overnight at 4 °C. This step was followed by incubation with a horseradish peroxidase-antibody conjugate (Abcam) directed against the primary antibody for 1 h. The membranes were then treated with chemiluminescent reagents (luminol and enhancer) and exposed to light-sensitive film. Images of these films were captured, and protein bands were quantified by using computerized image analysis (Gel Doc 2000, BioRad, Hercules, CA).

Primary antibodies used included Mouse monoclonal anti-rat MHC_slow_ antibody (ab11083, Abcam, UK); Mouse monoclonal anti-rat MHC_2A_ antibody (F18, DSHB, University of Iowa, USA); Rabbit polyclonal anti-calpain1 large Subunit antibody (ab28258, Abcam, UK); Rabbit polyclonal anti-calpain2 large Subunit antibody (ab39165, Abcam, UK); Mouse monoclonal anti-αII spectrin antibody (sc-46696, Santa Cruz, USA); Rabbit polyclonal anti-MAFbx antibody (ab74023, Abcam, UK); and Rabbit polyclonal Anti-MuRF1 antibody (ab172479, Abcam, UK) and mouse polyclonal anti-GAPDH antibody (Good Science, Shanghai, China). Secondary antibodies consisted of Horseradish peroxidase conjugated goat anti-mouse IgG antibody and Horseradish peroxidase conjugated goat anti-rabbit IgG antibody.

### Histology experiments

Serial cryosections were cut from the frozen biopsies (8 μm thick). Microscope slides were rehydrated in phosphate buffer (PBS) and subsequently blocked with phosphate buffer containing 1% bovine serum albumin (PBS-1%BSA). Cryosections were incubated with antibody for MyHC_slow_ (1:100, ab11083, Abcam) and for MyHC_fast_ (1:100, ab51263,Abcam) followed by appropriate fluorescent-labeled secondary antibodies (Invitrogen). Fibers were visualized by an antibody reactive to laminin (1:100, ab11575, Abcam). Following each incubation, cryosections were washed four times for 5 min with PBS. The cross-sectional area (CSA) of diaphragm muscle fiber was determined from a sample of 25–30 fibers of each type per animal (eight animals per group). Sections were analyzed with a Leica DM6000B microscope (Leica Application Suite). CSA was calculated by ImageJ software.

### Statistical analysis

Continuous data are reported as mean and standard error and were compared using unpaired Student’s *t* test or Wilcoxon rank-sum test after testing for normal distribution (Shapiro-Wilk). When comparing multiple independent means, a one-way analysis of variance (ANOVA) was first performed to confirm a difference across all groups prior to comparison of individual means. Results were considered significant if *p* values were less than 0.05. Statistical analysis was performed with the SPSS statistical package (v.19).

## Results

### Systemic and biologic response to mechanical ventilation

Blood pressure was maintained within physiologic ranges during the course of mechanical ventilation and did not differ significantly between groups (*p* > 0.05). Blood gas analysis after 24 h of MV is shown in Table 1. Initial body weights in CMV and CMVC were not significantly different from CON group (*p* > 0.05; Table 1). The total pentobarbital dose was similar in the two ventilated groups (98.37 ± 13.05 mg vs. 100.62 ± 12.79 mg in CMV and CMVC, respectively).

**Table 1:** Initial body weight, arterial blood pressure, and blood gas data in CON, CMV, and CMVC

	N	Initial body weight, g	pH	PaCO_2_, mmHg	PaO_2_, mmHg	Heart rate, bpm	Blood pressure, mmHg
CON	8	363 ± 21	NA	NA	NA	343 ± 27	123 ± 12
CMV	8	353 ± 22	7.41 ± 0.03	41.5 ± 2.90	95.4 ± 8.18	346 ± 26	123 ± 12
CMVC	8	368 ± 27	7.40 ± 0.02*	39.4 ± 3.16*	93.4 ± 8.13*	350 ± 22	121 ± 11
F	–	0.78	–	–	–	0.97	0.28
*p*	–	>0.05	–	–	–	>0.05	>0.05

Values are mean ± SD

*CON* control animals, *CMV* controlled mechanical ventilation, *CMVC* CMV treated with calpeptin, *F* the value of ANOVA in the three groups, *p* values are for ANOVA

*p > 0.05 for CMVC vs. CMV

### Diaphragm muscle myosin heavy chain content and cross-sectional area

**Fig. 1:**
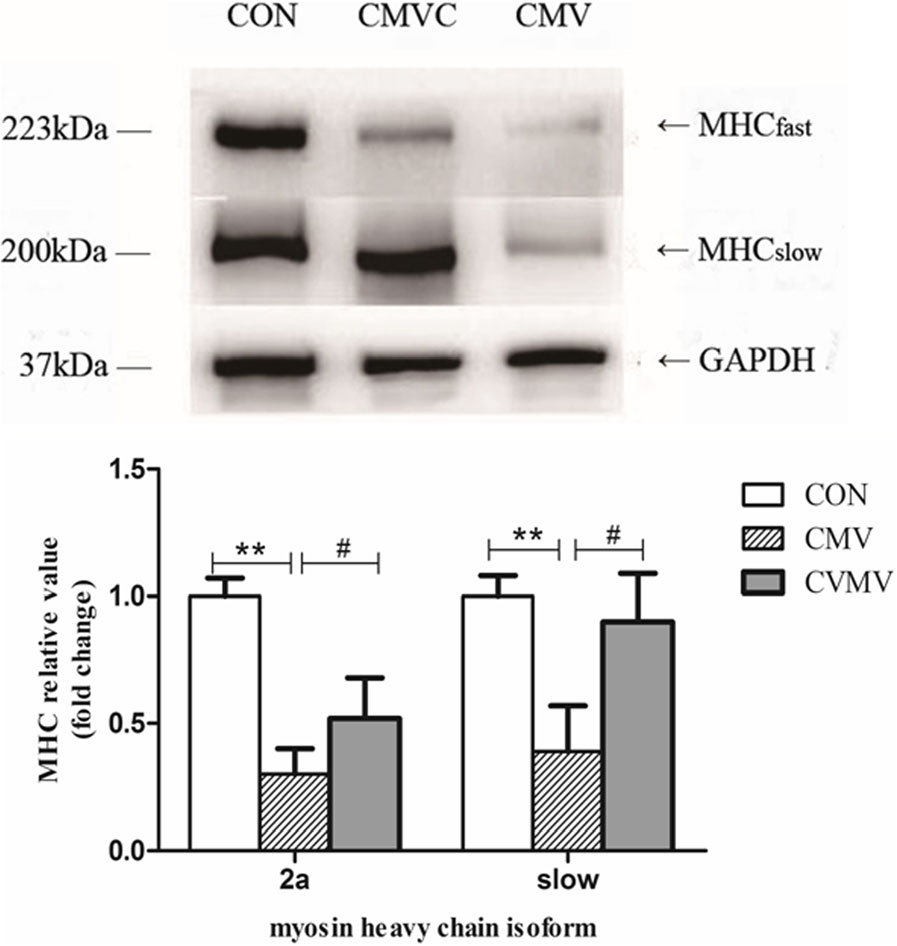
Western blot analyses of the two isoforms of the myosin heavy chain. GAPDH used as a reliable internal control. The results are expressed as a ratio of control group. *CON* control animals, *CMV* controlled mechanical ventilation, *CMVC* CMV treated with calpeptin. CMV rats received corresponding volumes of vehicle. **p < 0.01, #*p* < 0.05

**Fig. 2:**
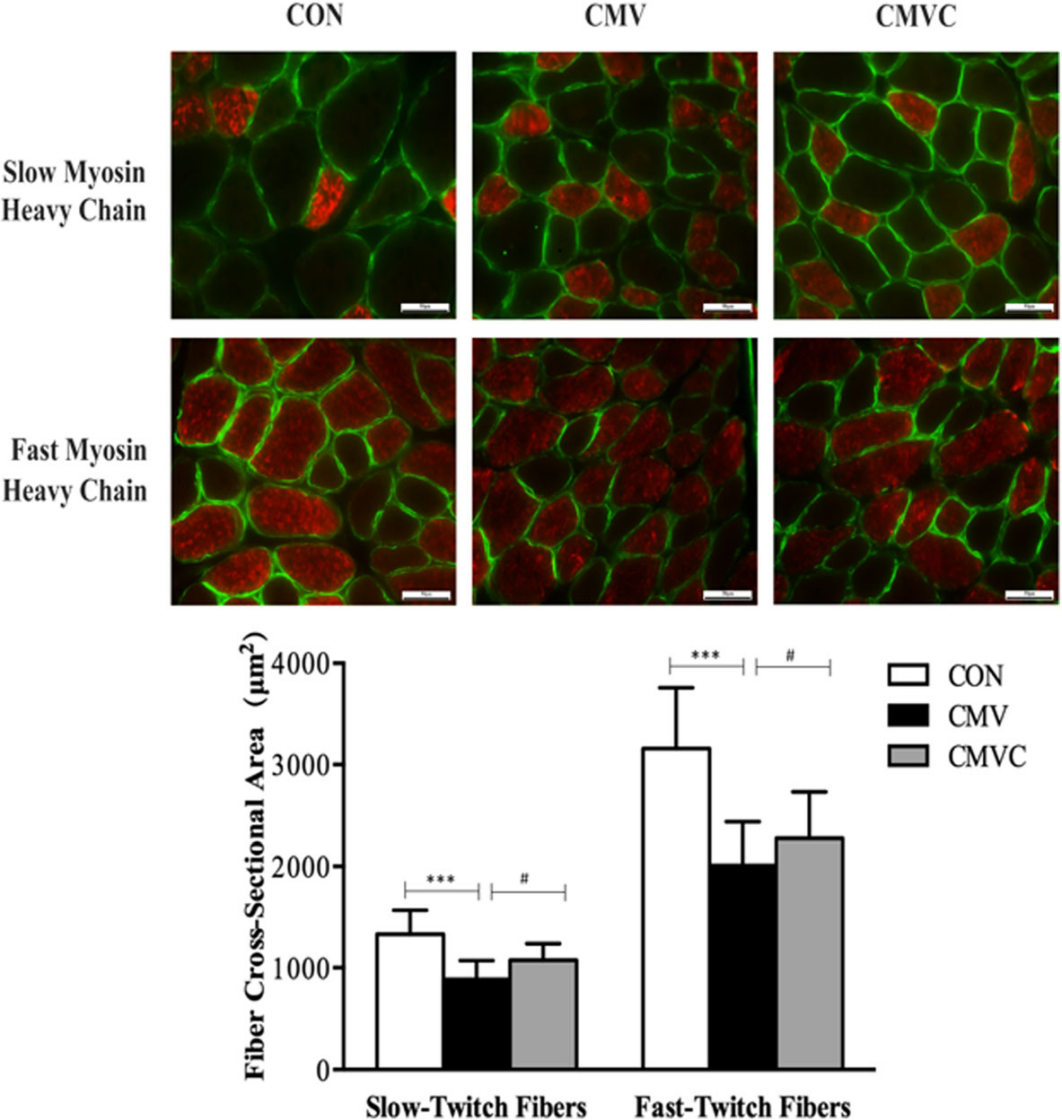
Diaphragm muscle cross-sectional area (CSA) in diaphragm skeletal muscle myofibers. In each of the sections, fibers reacting with the antibody appear *red*, whereas fibers not reacting with the antibody appear *black*. All fibers are outlined by an antibody reactive to laminin (ab11575, Abcam) which appear *green. Scale bars* represent 50 μm. *CON* control animals, *CMV* controlled mechanical ventilation, *CMVC* CMV treated with calpeptin. Each *bar* represents 200–240 fibers. CMV rats received corresponding volumes of vehicle. ***p < 0.001, #*p* < 0.05

CMV for 24 h resulted in a significant reduction in MHC_slow_ and MHC_2A_. In calpeptin-treated animals, both MHC_slow_ and MHC_2A_ density were significantly higher compared with CMV (Fig. 1). As shown in Fig. 2, the CSA of diaphragmatic muscle fibers both MHC_slow_ and MHC_fast_ was decreased significantly by 34 and 37% in 24 h of CMV compared with CON group, respectively. However, administration of the calpain inhibitor calpeptin significantly prevented loss of CSA of MHC_slow_ and MHC_fast_ compared with CMV, indicating that calpeptin reduced ventilator-induced diaphragm atrophy.

### Calpain activation

**Fig. 3:**
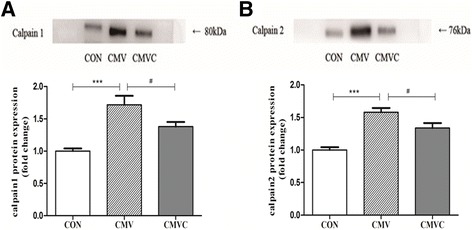
Protein levels of both calpain1 and calpain2 in the diaphragm. The images above the histograms in panels **a** and **b** are representative Western blots of data from the three experimental groups. The results are expressed as a ratio of control group. *CON* control animals, *CMV* controlled mechanical ventilation, *CMVC* CMV treated with calpeptin. CMV rats received corresponding volumes of vehicle. ***p < 0.001, #*p <* 0.05

**Fig. 4:**
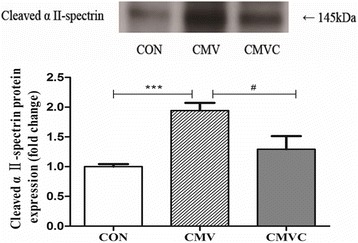
Levels of the 145 kDa α-II spectrin breakdown product in diaphragm. 145 kDa is a α-II -spectrin breakdown product that is specific to calpain1/2 cleavage of intact αII-spectrin. The images above the histograms are representative Western blots of data from the three experimental groups. The results are expressed as a ratio of control group. *CON* control animals, *CMV* controlled mechanical ventilation, *CMVC* CMV treated with calpeptin. CMV rats received corresponding volumes of vehicle. ***p < 0.001, #*p <* 0.05

Calpain activation was assessed in diaphragm homogenates by the protein expression of calpain1 and calpain2 and calpain cleavage fragment. The 145-kDa protein fragment is specific to the calpain-dependent cleavage products of αII-spectrin. As shown in Fig. 3, 24 h of CMV resulted in a significant increase in calpain1 and calpain2 protein levels in the diaphragm compared to CON. In line with this, the protein level of αII-spectrin breakdown product in the diaphragm was significantly increased in CMV group compared with the CON (Fig. 4).

Treatment with the calpain inhibitor calpeptin significantly reduced protein expression of calpain1 and calpain2 in CMV diaphragm (Fig. 3). In line with this, calpeptin significantly reduced αII-spectrin in ventilated rats (Fig. 4).

### E3-ligase content

**Fig. 5:**
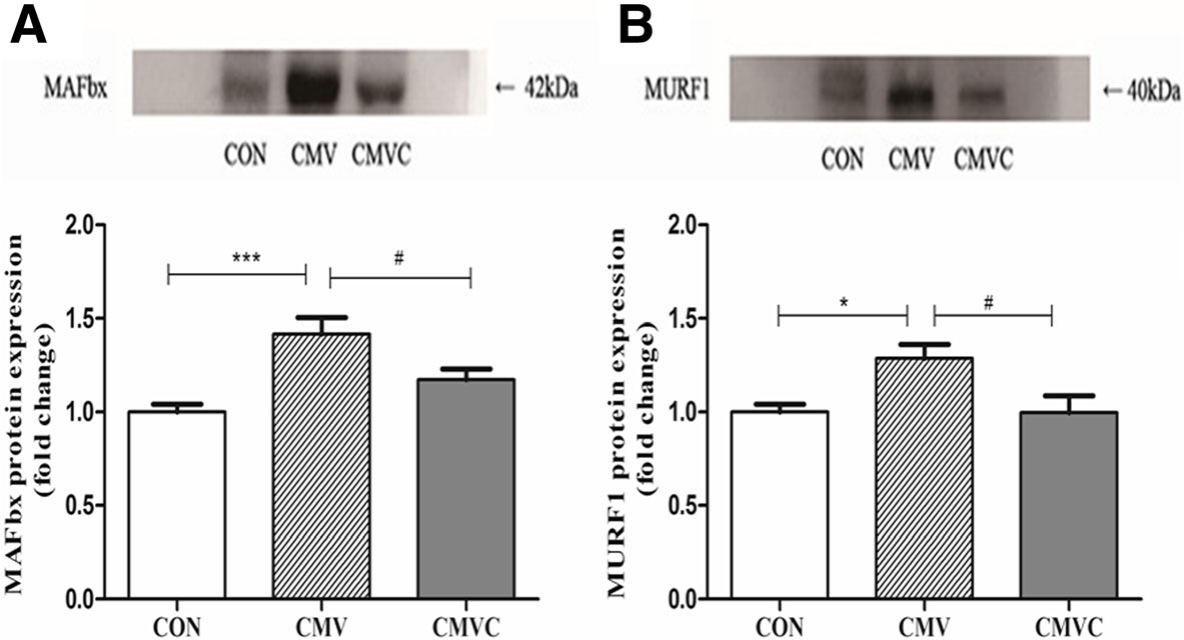
Protein levels of both MAFbx and MURF1 in the diaphragm. The images above the histograms in panels **a** and **b** are representative Western blots of data from the three experimental groups. The results are expressed as a ratio of control group. *CON* control animals, *CMV* controlled mechanical ventilation, *CMVC* CMV treated with calpeptin. CMV rats received corresponding volumes of vehicle. *p < 0.05, ***p < 0.001, #*p <* 0.05

Compared with CON, CMV rats exhibited higher levels of both MAFbx and MuRF1 protein expression (Fig. 5) in the diaphragm. Both MAFbx and MuRF1 protein levels were lower in the diaphragm after treatment with calpeptin (Fig. 5).

## Discussion

This study investigated the effects of calpain inhibition on ventilator-induced diaphragm atrophy and activation of proteolytic pathways. The main findings of our study can be summarized as follows: (1) calpain inhibitor calpeptin attenuates CMV-induced loss of myosin and CSA in the diaphragm muscle; (2) calpeptin treatment prevents the upregulation of calpain and the increase of calpain cleavage products; (3) calpeptin treatment reduces CMV-induced protein expression of the E3-ligases MAFbx and MuRF1. Collectively, our data indicate that calpain play an important role in CMV-induced diaphragm atrophy. Moreover, this study shows that inhibition of calpain activation prevents CMV-induced muscle atrophy.

Calpeptin is a dipeptide aldehyde and commonly used calpain inhibitor. Because calpeptin is membrane permeable, it is capable to penetrate into the cytosol of cells. There it binds to the critical cysteine residue in the active site of calpains [19]. Of note, Tsujinaka et al. showed that calpeptin was the most potent among synthesized inhibitors in terms of preventing the Ca^2+^-ionophore induced degradation of actin-binding protein and platelet talin in intact platelets. Furthermore, after 30-min incubation with intact platelets, calpeptin completely abolished calpain activity in platelets [20].

Previous studies in rats have demonstrated that CMV is associated with activation of the UPP, loss of contractile protein, and diaphragm dysfunction [5, 7]. For example, 12 h of CMV resulted in a decrease in force generation, promoted diaphragm fiber atrophy, and increased transcription of the muscle-specific E3-ligases MuRF1 and MaFbx in the diaphragm [5]. This is in line with our data, showing that 24 h of CMV induces loss of myosin and CSA in both slow and fast diaphragm fiber and an increase in protein expression of MuRF1 and MaFbx in the rat diaphragm. Recent studies in humans established that activation of the UPP is associated with loss of muscle protein in the diaphragm of patients receiving CMV [3, 8]. Levine and colleagues [8] reported that CMV for 18 to 69 h resulted in large decreases (i.e., 60%) of slow MyHC, fast MyHC, and a-actin in the diaphragm of brain dead organ donors. Also, critically ill patients receiving MV display atrophy and activation of UPP in both slow- and fast-diaphragm muscle fibers [3].

Although the UPP is activated during CMV, experimental inhibition of this proteolytic system has only been partially effective in preventing diaphragm muscle dysfunction [5, 21]. An explanation for this limited success is that other proteolytic systems act upstream of the UPP. Calpains are known to cleave myofilaments from the sarcomeric lattice [22] and thereby enable the UPP to degrade muscle protein into small peptides [23]. Immunolocalization studies showed that the calpains are concentrated in the Z-disk and I-band areas of the myofibril [22]. The Z-disk acts as anchor and mechanically links thin filaments from one sarcomere to the next along the myofibril [24]. One calpain substrate is the giant sarcomeric protein titin, which contains high-affinity calpain binding sites where the protein can be cleaved [25, 26]. Ex vivo experiments have shown that treatment of myofibrils from normal muscle with μ-calpain or m-calpain closely mimic structural disintegration as observed in rapidly atrophying [11, 22].

Several previous studies have evaluated the effect of calpain inhibition on preventing skeletal muscle atrophy [13, 27, 28]. For instance, inhibition of the calpain activity preserves sarcomere structure and attenuated the development of muscle weakness in hindlimbs of mice after 14 days of suspension [27]. During a 10-day unloading period of hindlimbs in mice, transgenic expression of calpastatin was also effective in reducing muscle atrophy by 30% [28].

Today, only one study assessed the role of calpain in the development of diaphragm dysfunction during CMV [12]. The authors showed that 12 h of MV of rats resulted in calpain activation in the diaphragm which was accompanied by diaphragm fiber atrophy and reduced force-generating capacity. This is in line with our data of 24 h of CMV. Treatment with the calpain inhibitor SJA-6017 in that study prevented the development of diaphragm fiber atrophy and weakness. Notably, diaphragm fiber force-generating capacity was expressed as specific force, i.e., tension normalized for cross-sectional area. So, the prevention of diaphragm weakness by calpain inhibition is only partially explained by attenuation of fiber atrophy, as calpain inhibition also increased specific force-generating capacity, independent of fiber size. The data of our study show that calpain inhibition prevented the loss of the major contractile protein myosin heavy chain. So, calpain inhibition not only prevents the loss of fiber size, but as our data show, also the loss of fiber contractile protein content. If the loss of contractile protein content exceeds the loss of fiber size, this would explain that calpain inhibition is able to prevent reduction of specific force-generating capacity in CMV animals. Interestingly, Nelson and colleagues also found that treatment with a calpain inhibitor attenuated activation of caspase-3 and vice versa [12]. This indicates that a regulatory cross talk exists between calpains and caspases for the modulation of CMV-induced diaphragmatic muscle atrophy. Our data suggest that a complimentary cross talk exists between the calpain system and the UPP. Because we showed that treatment with the calpain inhibitor calpeptin not only prevents calpain activation during CMV but also reduces the protein expression of the muscle-specific E3 ligases MuRF1 and MAFbx. To complete the circle of cross talks between calpain, caspase-3, and the UPP, a recent study showed that treatment of CMV rats with the proteasome inhibitor bortezomib partially protected the diaphragm against the development of weakness, most probably by indirect inhibition of caspase-3 [21]. The exact mechanism of these cross talks should be further investigated.

It is clear that prolonged MV results in diaphragmatic atrophy and contractile dysfunction in both animals and humans [2, 29]. However, in clinical settings, diaphragm dysfunction can also be caused or exacerbated by other factors, such as sepsis, malnutrition, and medicine. Sepsis is a major cause of mortality and long-term morbidity in ICU patients. Diaphragm dysfunction is strongly associated with sepsis [30]. Limited information exists concerning the combined impact of sepsis and MV on diaphragm function in humans or animals. In septic rats, Ebihara et al. [31] reported that 4 h CMV prevents diaphragmatic sarcolemmal injury and improves force-generating capacity of the diaphragm. However, Maes et al. [32] used an experimental rat model to simulate ICU settings in which patients with sepsis are MV in response to the development of multiple organ failure. In this protocol, CMV was applied 12 h after the initiation of sepsis. They found that 12 h of CMV in septic animals lead to worsening of diaphragm contractile dysfunction compared with CMV or sepsis alone but they found no exacerbation of muscle fiber atrophy. Future studies are needed to be investigated the interaction between the effects of mechanical ventilation and sepsis.

### Clinical implications

Numerous data exists, even in humans [2, 3], that CMV could have deleterious effect on diaphragm structure and function. The most potential targets of pharmacological actors to prevent this seem to be upstream of the UPP. The current data, together with previous data in the literature, provides evidence that calpain inhibition might be an effective therapeutic target in patients with MV-induced muscle weakness. However, to our knowledge, no clinical study has reported the use of calpain inhibitors yet. Calpastatin is an endogenous calpain inhibitor and might therefore be the more safe option to start studies with. As in the current experimental study, we aimed to proof a role for calpains in the development of CMV-induced diaphragm atrophy; we administered the first dose of calpain inhibitor 2 h before the start of CMV. This strategy will not be feasible to implement in current daily clinical practice because diaphragm dysfunction is mostly recognized after a prolonged time of CMV. Nevertheless, recent studies have demonstrated that diaphragm dysfunction may also develop during elective surgery [33, 34]. In such patients prophylactic treatment may be of clinical relevance.

## Conclusions

The current study showed that 24 h of CMV induced the activation of the calpain and UPP in the diaphragm. Specifically, treatment with a calpain inhibitor attenuated MV-induced muscle atrophy and calpain activation. Furthermore, calpain inhibition lead to reduced expression of muscle-specific E3-ligases suggesting a cross talk with the UPP. These finding provide more evidence that calpains are potential therapeutic target for treatment of CMV-induced diaphragm dysfunction.

## Abbreviations

CMV: Controlled mechanical ventilation
CMVC: CMV treated with calpeptin
CON: Control animals
CSA: Cross-sectional area
GADPH: Glyceraldehyde-3- phosphate dehydrogenase
MHC: Myosin heavy chain
UPP: Ubiquitin-proteasome pathway
VIDD: Ventilator-induced diaphragm dysfunction
